# Quality of life as an outcome variable in the management of advanced cancer

**DOI:** 10.4103/0971-5851.76194

**Published:** 2010

**Authors:** Manisha Bisht, S. S. Bist, D.C. Dhasmana, Sunil Saini

**Affiliations:** *Department of Pharmacology, Himalayan Institute of Medical Sciences, HIHT University, Jolly-grant, Dehradun – 248 140, Uttarakhand, India*; 1 *Department of ENT, Himalayan Institute of Medical Sciences, HIHT University, Jolly-grant, Dehradun – 248 140, Uttarakhand, India*; 2 *Department of Oncology, Himalayan Institute of Medical Sciences, HIHT University, Jolly-grant, Dehradun – 248 140, Uttarakhand, India*

**Keywords:** *Advanced cancer*, *cancer pain*, *quality of life*

## Abstract

**Background and Objective:**

Though well recognized in the West, palliative care and quality of life are relatively newer concepts in a developing country like India. The aim of this study was to assess the effect of palliative care on pain and quality of life and to identify the association between the two.

**Study Design:**

Observational prospective study.

**Materials and Methods:**

Patients with advanced cancer, receiving palliative drug therapy, were recruited from a tertiary care hospital. City of Hope Medical Center Quality of Life Survey and visual analog scale (VAS) were used to assess the quality of life and cancer pain severity, respectively.

**Results:**

A total of 100 patients were included in the study. Palliative drug therapy produced a significant reduction in pain scores expressed as mean ± SD in VAS [7.13 ± 2.2 vs. 2.62 ± 2.1 (*P*<0.001) after 1 month in 93 patients; 7.06 ± 2.1 vs. 2.47 ± 2.1 (*P*<0.001) after 1 month and 2.02 ± 1.9 (*P*<0.001) after 2 months in 51 patients]. Also, significant improvement in the quality of life scores [919.78 ± 271.3 vs. 1280.65 ± 306.8 (*P*<0.01) after 1 month in 93 patients; 950.39 ± 238.2 vs. 1336.67 ± 291 (*P*<0.01) after 1 month and 1405.49 ± 368.3 (*P*<0.01) after 2 months in 51 patients] was obtained. There was a high correlation between the average change of pain intensity and quality of life scores (*r*= −0.53, *P*<0.02). Overall, a reduction in pain resulted in significant improvement in the quality of life (*P*<0.001).

**Conclusion:**

This study emphasizes the role of palliative care and, more importantly, pain management in improving the quality of life of advanced cancer patients.

## INTRODUCTION

Fifty percent of cancer patients suffer an from advanced stage of disease that unfortunately is not responsive to curative treatment; however, with palliative care, even these patients may live for years. Patients with advanced cancer may suffer from innumerable symptoms like pain, anorexia, nausea or vomiting, depression, dyspnea, malnutrition, dry mouth, anxiety, skin problems and sleeplessness.[1] The only available management for such patients is palliative care, which focuses primarily on pain relief.[2] World Health Organization (WHO) defines palliative care as “the total active care of the patient whose disease is not responsive to curative treatment”. Palliative care is concerned primarily with controlling symptoms, managing side effects and supporting overall quality of life when cure or control of the cancer is no longer believed to be possible.[3] More than 80% of patients with cancer develop pain before death. Control of pain, of all other symptoms, is of utmost importance. Traditionally, the success of cancer treatment has been measured by end points suchas patient survival or disease-free survival and assessing the tumor response, by change in tumor volume. Increasingly, researchers are faced with situations where patientsmay not gain benefits in terms of traditional end points. For these patients, quality of life assessment is important, especially when prolongation of survival is not expected. Quality of life is now recognized as an end point of secondary importance next only to survival.[4] Though well established in the West, the concept of palliative care is new to India, having developed only in the past few years. Since control of pain is most important than control of any other symptom, we have seen the association of pain intensity and quality of life. Pain is strongly associated with quality of life and has been suggested as an important indicator for quality of life of patients with cancer.[4] Several instruments have been developed to assess the quality of life in patients with cancer pain. Among them, the City of Hope Medical Center Quality of Life Survey is a validated, accurate and internationally accepted survey instrument.[5] The use of such a questionnaire allows evaluation of outcomes of palliative care and health-related quality of life and leads to better understanding of patient expectations. The aim of this study was to evaluate the outcome of palliative care in terms of improvement of quality of life of patients and correlate it with pain improvement.

## MATERIALS AND METHODS

The study was conducted in the Oncology Clinics of a tertiary care teaching hospital. It was an observational cohort prospective/follow-up study of patients with advanced cancer, undergoing palliative drug therapy. A total number of 100 advanced cancer patients were included in the study. Approval for the study was obtained from the hospital’s institutional research committee. After informed consent was obtained, patients with advanced cancer were recruited and followed up monthly for 2 months. Upon recruitment, demographic data, relevant medical history and previous drug therapy were all recorded. During the 2-month period, patients received medical treatment as judged necessary by responsible physicians and no effort was made to alter or modify the course of treatment. The effect of the palliative care was evaluated on mainly two parameters, namely, pain and quality of life. The instruments used to evaluate the patients included visual analog scale (VAS) for pain and the City of Hope Medical Center Quality of Life Survey for quality of life measurement. VAS is a scale presented as a horizontal row of equidistant numbers from 0 to 10, with the ratings given as “no pain” at 0 and “pain as bad as you can imagine” at 10.The quality of life instrument used in the study was the City of Hope Medical Center Quality of Life Survey. It is a multidimensional instrument developed to evaluate quality of life as a measure of pain management outcome in individual patients.[5] It consists of 28 VAS items with word extremes as anchors at each end. Item scores range from 0 to 100. This tool is modeled after the quality of life instruments tested by Padilla and Grant,[6] and includes items in the areas of psychological and physical well-being, general symptom control, specific symptom control, and social support. This instrument has undergone testing with cancer patients to explore reliability and sensitivity features to analgesic intervention.[5] Patients completed the instruments in the hospital setting. Although the questionnaire was designed as a self-reported scale, the high illiteracy rates in our population did not allow for effective use of self-reported questionnaire. Those patients having difficulty completed the questionnaire with assistance from the researcher. The researcher read out exactly what was written, did not change the items, and did not make any additional explanation and recorded their verbal responses. Data at the study entry, and 1 and 2-month follow-up were used for this analysis. Data were analyzed using the Statistical Package for Social Sciences (SPSS). Descriptive statistics were used for demographic characteristics and presented as percentage and mean±SD, where appropriate. Dependent *t*-test was used for analyzing the difference of pain and quality of life scores at baseline and subsequent follow-up. The Pearson correlation coefficient was used to analyze the association between the mean changes in pain levels and mean changes in quality of life scores. Significance for all analyses was taken at the 5% level. Changes of pain scores at baseline and follow-up were compared against the change of quality of life scores by analysis of variance.

## RESULTS

**Figure 1:**
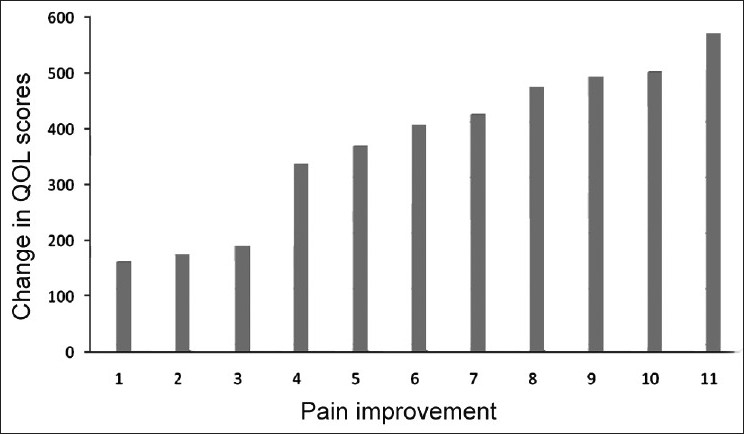
Average change of Quality of Life (QOL) scores at different levels of average change of pain scores from baseline

A total of 100 patients were enrolled for the study. One-month follow-up of only 93 patients was available. Three patients expired and four were lost to follow-up. Only 51 patients were available for 2-month follow-up, as most of the patients were receiving only symptomatic palliative therapy and were mostly provided home care, hence lost for follow-up in the subsequent months. Table 1 shows the demographic profile of the patients. Out of 100 patients, 60 (60%) were men and 40 (40%) were women. The mean±SD age was 52.57±13.02 years (range 13–80), and the mean±SD Karnofsky index was 64.44±12.39 (range 40–90). The most common cancer presented in our study was lung cancer (34%), followed by gastrointestinal tract (25%), breast cancer (10%), head and neck cancer (9%), primary unknown tumor (8%), gynecological (7%) and other tumors (5%). At the time of diagnosis, 62 patients had distant metastasis, 80 had locally advanced disease and 7 had recurrent tumor. The common symptoms experienced by the patients included pain, weakness/fatigue, anorexia, insomnia, nausea/vomiting, dyspnea, constipation and cough. Pain was the most frequent symptom occurring in 95% of patients. At study entry, 62% of the patients reported severe pain as compared to only 3 and 1% at 1 and 2 months, respectively. The pain was controlled in all patients mainly with drugs according to World Health Organization (WHO) analgesic guidelines. Non-narcotic analgesics (WHO level 1) were used by 89.47% of the patients in pain. Weak opiates (WHO level 2) and strong opiates (WHO level 3) were administered in only 65.26 and 6.32% of the patients in pain, respectively. Apart from analgesics, otherdrugslike antiemetic, proton pump inhibitors, systemic antibiotics, antiasthmatics and vitamin supplements were among the most frequently used medications. Overall, the patients received 8.7±3.8 (mean±SD) drugs on an average during the observation period of 2 months. At the time of the study,55% of patients received palliative chemotherapy. Palliative surgery was done in 23% and radiotherapy was received by 23% of the patients. The mean pain intensity significantly decreased from baseline at both 1 and 2 month follow-up. In 93 patients, the mean±SD pain intensity at 1 month indicated a 63% reduction from baseline [7.13±2.2 vs. 2.62±2.1 (*P*<0.001)], whereas in 51 patients, there was 65% reduction in mean pain intensity [7.06±2.1 vs. 2.47±2.1 (*P*<0.001)] after 1 month and 71% [7.06±2.1 vs. 2.02±1.9 (*P*<0.001)] after 2 months. Similarly, significant improvement in the quality of life scores [919.78±271.3 vs. 1280.65±306.8 (*P*<0.01) after 1 month in 93 patients; 950.39±238.2 vs. 1336.67±291 (*P*<0.01) after 1 month and 1405.49±368.3 (*P*<0.01) after 2 months in 51 patients] was observed [Table 2]. There was a high correlation between the average change in pain severity and quality of life total scores (*r*= −0.53, *P*<0.001). Overall, a reduction of pain results in statistically significant improvement in quality of life [Table 3, Figure 1].

**Table 1:** Baseline demographic and baseline characteristics of patients attending oncology clinics

	No. of patients (*N*=ioo)
Sex distribution	
Male	60
Female	40
Age distribution	
Mean±SD	52.57±13.02
Range	13–80 years
Cancer distribution	
Lung	33
Gastrointestinal	28
Breast	10
Head and neck cancer	9
Primary unknown tumor	8
Gynecological	7
Others	5
Occupation	
Service	13
Self-employed	23
Housewife	38
Others	26
Habits	
Smoker	56
Alcoholics	49
Both	45
Education	
Illiterate	45
Primary school	34
Secondary high school	10
University	11
Karnofsky performance status	Mean±SD: 64.44±12.39 Range: 40–90
≤70	15
>70	85
Tumor burden	
Distant metastasis	62
Locally advanced	80
Recurrence	7
Other palliative treatment	
Chemotherapy	55
Surgery	23
Radiotherapy	23

**Table 2:** Effect of palliative drug therapy on pain scores and quality of life in patients with advanced cancer over a period of 2-month follow-up

	VAS$ (mean±SD)	Quality of life# (mean±SD)
Baseline (*n*=93)	7.13±2.2	919.78±271.3
1 Month (*n*=93)	2.62±2.1***	1280.65±306.8**
Baseline (*n*=51)	7.06±2.1	950.39±238.2
1 Month (*n*=51)	2.47±2.1***	1336.67±291**
2 Months (*n*=51)	2.02±1.9***	1405.49±368.3**

** *P*<0.01

*** *P*<0.001 versus 0 month baseline values

$ VAS = Visual analog scale (0, no pain; 10, maximum pain). Decrease in score implies reduction in pain

# Quality of life: Quality of life index (0, minimum; 2800, maximum). Increase in score denotes improvement in quality of life

**Table 3:** Average change of quality of life at all levels of change of pain scores reported at 1 month compared with baseline (*n*=93)***

Change in pain score*	Mean difference (SD) of quality of
	life scores**
0	160 (82.8)
−1	175 (91.9)
−2	190 (115.9)
−3	335.5 (118.3)
−4	367.2 (194.9)
−5	405.86 (292.9)
−6	424 (174.9)
−7	475 (159.8)
−8	493.85 (210.14)
−9	500 (192.7)
−10	572.5(72.3)

* Visual analog scale: Higher score means worsened pain

** Quality of life scores = Higher score means improved quality of life;

*** Analysis of variance (ANOVA) test resulted in *P* value <0.001 in quality of life scores, at different levels of pain classified as no change, little (−1 to −3), moderate (−4 to −6), and much (>−7) reduction

## DISCUSSION

Patients suffering from advanced cancer have numerous symptoms, which require comprehensive treatment. Palliative care aims to improve the quality of life in these patients by adequate symptom management. Pain was the most common symptom experienced by the patients in our study. This was in accordance to study done in a palliative care clinic in India, where the prevalence of pain was nearly 90%.[7] Advanced cancer patients frequently receive polypharmacotherapy as multiple symptoms need to be treated. In this study, patients received 8.7±3.8 (mean±SD) drugs on an average during the observation period of 2 months. One study had documented that patients may receive 2.43 supportive drugs on average.[8] The higher number of drugs in our study may be attributed to the fact that we had included the drugs used for palliative chemotherapy also in the study. Assessing treatment outcomes in palliative care is difficult. The outcome measure in our study was quality of life. WHO has clearly mentioned that quality of life is more appropriate outcome variable for evaluating the efficacy of palliative care. In this study, we have highlighted the favorable effect of palliative drug therapy on quality of life. Previous studies elsewhere have already documented that improved treatment of symptoms is associated with enhanced quality of life and patient satisfaction.[9,10] Inclusion of quality of life in cancer research is common in the West but a few studies have been conducted from a developing country like India. This is the first study done in our settings to evaluate the effect of palliative care. Pain has been cited as the key component of quality of life;[11] therefore, we have used the quality of life tool which is sensitive to capture the effects of pain management overtime. Many instruments have been developed to measure the quality of life of cancer patients, but few focus on cancer-related pain.[12] The reliability and validity of the questionnaire used in our study have been validated previously.

A significant decrease in overall pain intensity parallel to quality of life improvement was achieved in the study but was far from complete. It is globally recognized that there is a high prevalence of inadequate pain relief in a variety of clinical settings among cancer patients.[13] Our findings demonstrate a strong correlation between pain reduction and improvement of quality of life. Though it has been recognized that cancer-related pain diminishes patients’ quality of life, stillthere is dearth of studies where the effect of cancer pain on quality of life is directly evaluated. In one study, the change of pain scores was directly compared with quality of life of patients and it was demonstrated that pain had a significant effect on patients’ quality of life. Pain deterioration had slightly more impact on quality of life than pain improvement.[14] In our study, we did not find any deterioration in the pain intensity at follow-up in contrast to the above-mentioned study; so, the impact of pain deterioration on quality of life could not be compared. Although there was a significant improvement in quality of life of the patients parallel to the reduction in pain, there was still a scope of further improvement in both parameters, as pain was inadequately treated in our patients.

The present study has several limitations as well. First, the differentiation of pain and quality of life scores with respect to different sites of cancer were not analyzed. Also, the cause of pain, whether related to tumor or treatment, was not looked upon. Second, the quality of life scale was administered by the researcher, which may bias the results. Third, cultural difference between the developed country where the instrument was developed and the developing country where it was applied was not measured. Finally, these data come from a single institution study and therefore do not represent overall palliative care in hospitals throughout India. The shortcomings in the management of the patients in the study like incomplete pain relief are attributed to the lack of specialized palliative care services in our country as compared to the West. Further research is needed to find out the impact of comprehensive palliative care on quality of life. Studies done in the West have already demonstrated that the existence of a palliative care services results in improved standards of care.[15]

The present study demonstrates the evaluation of quality of life as an important tool even for a populations living in a developing country. Nonetheless, the present study represents the first report on quality of life assessment in Indian population. These data are important for health care workers and patients living in other developing countries who have limited access to health care.
